# Dr. William H. Vincent

**Published:** 1905-08

**Authors:** 


Dr. William H. Vincent, a graduate of the University of Buffalo, 1881, died at his home in Hinsdale, N. Y., June 12, 1905, aged 51 years.

